# Racial and Ethnic Disparities in Emergency Department Care and Health Outcomes Among Children in the United States

**DOI:** 10.3389/fped.2019.00525

**Published:** 2019-12-19

**Authors:** Xingyu Zhang, Maria Carabello, Tyler Hill, Kevin He, Christopher R. Friese, Prashant Mahajan

**Affiliations:** ^1^Department of Systems, Populations, and Leadership, University of Michigan School of Nursing, Ann Arbor, MI, United States; ^2^Department of Health Management and Policy, University of Michigan School of Public Health, Ann Arbor, MI, United States; ^3^Department of Biostatistics, University of Michigan School of Public Health, Ann Arbor, MI, United States; ^4^Department of Emergency Medicine, University of Michigan School of Medicine, Ann Arbor, MI, United States

**Keywords:** pediatrics, emergency department, disparity, racial/ethnic, trend

## Abstract

**Background:** There is an incomplete understanding of disparities in emergency care for children across racial and ethnic groups in the United States. In this project, we sought to investigate patterns in emergency care utilization, disposition, and resource use in children by race and ethnicity after adjusting for demographic, socioeconomic, and clinical factors.

**Methods:** In this cross-sectional study of emergency department (ED) data from the nationally representative National Hospital Ambulatory Medical Survey (NHAMCS), we examined multiple dimensions of ED care and treatment from 2005 to 2016 among children in the United States. The main outcomes include ED disposition (hospital admission, ICU admission, and in hospital death), resources utilization (medical imaging use, blood tests, and procedure use) and patient ED waiting times and total length of ED stay. The main exposure variable is race/ethnicity, categorized as non-Hispanic white (white), non-Hispanic black (Black), Hispanic, Asian, and Other. Analyses were stratified by race/ethnicity and adjusted for demographic, socioeconomic, and clinical factors.

**Results:** There were 78,471 pediatric (≤18 years old) ED encounters, providing a weighted sample of 333,169,620 ED visits eligible for analysis. Black and Hispanic pediatric patients were 8% less likely (aOR 0.92, 95% CI 0.91–0.92) and 14% less likely (aOR 0.86, CI 0.86–0.86), respectively, than whites to have their care needs classified as immediate/emergent. Blacks and Hispanics were also 28 and 3% less likely, respectively, than whites to be admitted to the hospital following an ED visit (aOR 0.72, CI 0.72–0.72; aOR 0.97, CI 0.97–0.97). Blacks and Hispanics also experienced significantly longer wait times and overall visits as compared to whites.

**Conclusions:** Black and Hispanic children faced disparities in emergency care across multiple dimensions of emergency care when compared to non-Hispanic white children, while Asian children did not demonstrate such patterns. Further research is needed to understand the underlying causes and long-term health consequences of these divergent patterns of racial disparities in ED care within an increasingly racially diverse cohort of younger Americans.

## Introduction

In 2010, the American Academy of Pediatrics (AAP) released a report reviewing the extant literature on racial/ethnic health and health care disparities among US children, concluding that care disparities were “extensive, pervasive, and persistent” (p. e979) (1). The report described disparities across multiple dimensions of health and health care, including all-cause mortality. Despite the strong conclusions drawn from the 111 articles reviewed, the authors noted several gaps in the broader literature. In studies that fell short of review requirements, the most common flaws included failing to analyze children separately from adults and analyzing all non-white racial/ethnic demographics as one population. Further, even among the studies included in the final review, nearly a quarter failed to adjust for likely confounders, such as other demographic and socioeconomic factors.

While some of these gaps have been addressed in recent years, research on health disparities in children lags behind that focused on adults (2, 3). This is especially concerning given that the racial and ethnic composition of American youth is changing rapidly, with the US Census Bureau projecting that more than half of US children will be non-white or Hispanic by the year 2020 (4). Given this major demographic shift within younger cohorts, thoroughly understanding the nature and trends of youth racial/ethnic health disparities represents a critical area of concern for both clinical practice and health policy.

To fill this knowledge gap in an important sector of the US health care system, we examined patterns in emergency department (ED) care outcomes for Black, Hispanic, and Asian children relative to non-Hispanic white children. We also analyzed factors that may contribute to care disparities, including patient demographic, socioeconomic, and clinical factors—all identified as primary drivers of health disparities in the broader literature. Our analysis aims to explore correlates of racial/ethnic disparities in emergency care and treatment using a nationally representative sample of US children.

## Methods

We conducted a cross-sectional study of ED data obtained from a multiyear, nationally representative survey carried out in the US. This study used pre-existing, de-identified data and was categorized as exempt by the University of Michigan's Institutional Review Board.

### Data Source and Study Population

The study population was derived from the National Hospital Ambulatory Medical Survey (NHAMCS) Emergency Department Subfile (NHAMCS-ED) between 2005 and 2016 (5). NHAMCS-ED is a multistage stratified probability sample of ED visits in the US, administered by the National Center for Health Statistics, Centers for Disease Control and Prevention. The NHAMCS-ED sample is collected from ~300 hospital-based EDs per year, randomly selected from roughly 1,900 geographic areas in all 50 states. The survey uses a standardized data collection form to capture detailed information from ~100 patients per hospital-based ED.

A total of 3,58,163 patient (weighted *N* = 1,560,846,342) visits from 3,764 hospital-based EDs were included in the analytic dataset. To restrict our sample to pediatric patients with a single documented race/ethnicity, we excluded patients with unknown or multiple races listed (*n* = 31,703; weighted *N* = 161,739,887) and those over the age of 18 (*n* = 247,989, weighted *N* = 1,065,936,835). This resulted in a final sample of 78,471 (Weighted *N* = 333,169,620) pediatric patients presenting to US EDs.

### Study Outcomes

The primary study outcome variables include the Emergency Severity Index (ESI), a five-level ED triage algorithm providing clinically relevant stratification of patients into five groups from 1 (most urgent) to 5 (least urgent) on the basis of visit acuity level and resource needs; ED disposition, specifically hospital admission and intensive care unit (ICU) admission; medical resource utilization (blood test, imaging, and other procedures; see Supplement Table 1 for a full list); waiting time (time between arrival and seeing a physician); and length of visit (time from arrival to discharge/disposition) for the ED encounter. Death outcomes include deaths in either the ED or hospital. The primary exposure variables for the analysis were a patient's racial/ethnic categorization. Race was predefined by NHAMCS as Asian, Black, white, or Other (including those who identify as American Indian/Alaska native and Native Hawaiian/Other Pacific Islander), and ethnicity was categorized as either Hispanic or non-Hispanic. From these categorizations we arrived at the following mutually exclusive groups for analysis: non-Hispanic Black (hereafter referred to as Black), Hispanic, Asian, Other, and non-Hispanic white (hereafter referred to as white), as the baseline for comparison. For simplicity, we refer to these as racial groups throughout the remainder of the manuscript; all non-white categories are considered racial minorities for the purposes of our analysis. Given the small sample size and heterogeneity of “Other,” we report data for this group in the tables but do not focus on them in our discussion.

For adjusted analyses, we included patient demographic variables (sex and age group); variables indicative of socioeconomic status, including residence type (private home, nursing home, homeless, or other) and insurance type (private insurance, Medicaid/CHIP, Medicare, uninsured, or other); mode of arrival (ambulance vs. not); day of the week; and time of arrival. We also included clinical variables, such as triage vital signs (body temperature, heart rate, diastolic blood pressure, and pain scale). Additional patient-level covariates included the primary reason for the ED visit (categorized by system-based symptom clusters because this information was available in the dataset for the entire patient population) and whether the patient had visited the ED within the past 72 h. We also included information on the US census region of the ED.

### Statistical Analysis

Population characteristics were described and compared among different racial groups. The proportion of each outcome variable among different racial groups and covariate groups were compared using χ-square tests. Multinomial logistic regression models were used to estimate the association between the ESI scores (categorical) and racial groups. Models were sequentially adjusted for demographic, socioeconomic, and visit/clinical variables. Multivariable logistic regression models were used to estimate the association between each binary outcome (hospital admission, ICU admission, death, blood test, medical imaging utilization, and procedure use) and the racial groups. These models were sequentially adjusted for demographic, socioeconomic, and visit/clinical variables; specifically, we adjusted for ESI scores to test for changes in the associations between racial group and binary outcomes.

Multivariable linear regression was used to test the association between the waiting time and length of visit (continuous variables) and racial groups after adjusting for other confounding variables. Because these two variables were not normally distributed, a log transformation was performed prior to the regression model. Models were sequentially adjusted for demographic, socioeconomic, visit/clinical variables, and ESI score.

Poisson regression was used to estimate the prevalence rate of disposition and resource utilization outcomes each year among the racial groups, adjusting for age, gender, and insurance type, with time modeled linearly as years since 2005. An interaction between the racial group and time was included in each model to test whether the trend in each outcome differed across racial groups.

The NHAMCS-ED dataset used in this analysis relies on imputation for missing data (5). Specifically, the survey uses hot deck-based single, sequential regression to impute 3-digit ICD-9-CM codes for certain items, such as age, sex, primary diagnosis, ED volume, and geographic region. For the remaining variables (for which missing values are not imputed in the NHAMCS-ED dataset) we analyzed missing data patterns using the MI procedure in SAS. All the variables were deemed Missing Completely at Random. The missing data for these variables were imputed with the median of the corresponding variables prior to generating the logistic regression models, multivariable linear regression models, and Poisson models. SAS (version 9.4) was used for analyses with α = 0.05 set as the statistical significance threshold. All confidence intervals are 95%.

## Results

During the 12-years study period between 2005 and 2016, NHAMCS collected data on 78,471 pediatric (≤18 years old) ED encounters, providing a weighted sample of 333,169,620 for analysis. The analysis was stratified by racial group, with the following proportions represented in the final analytic sample: white (52.1%), Black (24.0%), Hispanic (20.6%), Asian (2.0%), and Other (1.3%). Rates of uninsurance were highest for Hispanics (10.6%) and Blacks (8.4%) and lowest for Asians (6.3%) and whites (7.8%). Across racial groups, children between 1 and 6 years old accounted for the highest proportion of ED visits. In terms of symptoms, Black patients presented with the highest proportion of respiratory issues (20.9% of visits) and Hispanics presented with the highest proportion of digestive issues (15.6% of visits). The full set of ED pediatric patient characteristics stratified by race or ethnicity are presented in Table 1 and Supplement Table 2. All differences between groups were significant (*p* < 0.01).

**Table 1:** Baseline characteristics of patients presenting to the ED, stratified by race/ethnics, NHAMCS 2005–2016.

	**All *n* (%)**	**White *n* (%)**	**Black *n* (%)**	**Hispanic *n* (%)**	**Asian *n* (%)**	**Other *n* (%)**
	333,169,620	173,692,657 (52.1)	80,086,839 (24.0)	68,613,863 (20.6)	6,503,054 (2.0)	4,273,207 (1.3)
Male	172,897,072 (51.9)	90,601,735 (52.2)	40,677,155 (50.8)	35,788,268 (52.2)	3,496,132 (53.8)	2,333,782 (54.6)
Age						
0–<1	39,525,262 (11.9)	17,668,772 (10.2)	10,280,378 (12.8)	9,927,448 (14.5)	952,275 (14.6)	696,388 (16.3)
1–<6	114,713,519 (34.4)	55,276,342 (31.8)	28,425,637 (35.5)	26,595,323 (38.8)	2,887,704 (44.4)	1,528,512 (35.8)
6–<12	72,902,816 (21.9)	38,100,893 (21.9)	17,011,239 (21.2)	15,654,639 (22.8)	1,289,632 (19.8)	846,414 (19.8)
12–18	106,028,023 (31.8)	62,646,650 (36.1)	24,369,584 (30.4)	16,436,453 (24.0)	1,373,443 (21.1)	1,201,893 (28.1)
Residence type						
Private residence	319,702,310 (99.1)	166,366,985 (99.1)	76,879,363 (99.0)	66,034,405 (99.4)	6,289,858 (99.4)	4,131,700 (98.4)
Nursing home	419,284 (0.1)	186,213 (0.1)	94,569 (0.1)	124,861 (0.2)	13,641 (0.2)	0 (0.0)
Homeless	320,886 (0.1)	143,226 (0.1)	119,622 (0.2)	38,973 (0.1)	1,799 (0.0)	17,266 (0.4)
Other	2,054,522 (0.6)	1,198,103 (0.7)	529,544 (0.7)	250,041 (0.4)	25,572 (0.4)	51,263 (1.2)
Insurance type						
Private insurance	106,953,324 (33.9)	72,494,939 (44.0)	17,034,962 (22.7)	13,403,030 (20.6)	2,785,655 (45.6)	1,234,739 (31.1)
Medicare	3,066,983 (1.0)	1,625,044 (1.0)	757,276 (1.0)	636,444 (1.0)	24,913 (0.4)	23,308 (0.6)
Medicaid or CHIP	170,807,425 (54.2)	73,809,598 (44.8)	49,296,858 (65.6)	42,752,364 (65.8)	2,738,750 (44.8)	2,209,855 (55.6)
Uninsured	26,695,106 (8.5)	12,793,709 (7.8)	6,298,764 (8.4)	6,883,680 (10.6)	385,087 (6.3)	333,866 (8.4)
Other	7,616,926 (2.4)	4,181,455 (2.5)	1,764,992 (2.3)	1,323,063 (2.0)	177,780 (2.9)	169,636 (4.3)
Arrival by ambulance	19,797,958 (6.1)	9,896,740 (5.8)	5,592,751 (7.2)	3,524,421 (5.3)	473,467 (7.4)	310,580 (7.5)
Seen within last 72 h	9,948,261 (3.5)	4,975,333 (3.3)	2,299,231 (3.4)	2,234,071 (3.8)	220,758 (3.8)	218,866 (5.5)
Pain level						
No pain	82,904,253 (37.9)	39,358,670 (34.0)	22,649,468 (43.1)	17,757,196 (41.4)	1,964,514 (44.6)	1,174,405 (39.7)
Mild	34,275,302 (15.7)	18,904,680 (16.3)	7,751,433 (14.8)	6,497,999 (15.1)	702,518 (16.0)	418,672 (14.2)
Moderate	63,055,670 (28.8)	36,086,738 (31.1)	13,505,438 (25.7)	11,362,699 (26.5)	1,272,600 (28.9)	828,195 (28.0)
Severe	38,450,234 (17.6)	21,564,307 (18.6)	8,588,085 (16.4)	7,298,073 (17.0)	464,206 (10.5)	535,563 (18.1)
Temperature (C°)						
36–38	264,632,865 (84.0)	140,243,512 (85.5)	63,344,163 (83.6)	52,819,823 (81.2)	4,833,426 (78.4)	3,391,941 (83.7)
≤36	11,990,313 (3.8)	7,218,093 (4.4)	2,509,484 (3.3)	1,978,962 (3.0)	161,091 (2.6)	122,684 (3.0)
>38	38,485,798 (12.2)	16,631,739 (10.1)	9,927,444 (13.1)	10,216,631 (15.7)	1,171,313 (19.0)	538,671 (13.3)
Heart rate (BPM)						
≤90	115,436,813 (34.6)	63,708,888 (36.7)	27,813,844 (34.7)	20,825,030 (30.4)	1,796,717 (27.6)	1,292,333 (30.2)
90–100	43,881,682 (13.2)	24,463,373 (14.1)	10,233,172 (12.8)	8,036,223 (11.7)	679,903 (10.5)	469,010 (11.0)
100–110	37,041,723 (11.1)	20,330,558 (11.7)	8,977,997 (11.2)	6,649,624 (9.7)	713,344 (11.0)	370,199 (8.7)
110–120	34,718,454 (10.4)	17,778,029 (10.2)	8,239,891 (10.3)	7,661,983 (11.2)	542,102 (8.3)	496,448 (11.6)
>120	102,090,949 (30.6)	47,411,809 (27.3)	24,821,934 (31.0)	25,441,002 (37.1)	2,770,988 (42.6)	1,645,217 (38.5)
DBP						
<60	134,026,805 (40.2)	72,642,858 (41.8)	32,263,768 (40.3)	25,319,628 (36.9)	2,368,029 (36.4)	1,432,522 (33.5)
60–80	144,151,078 (43.3)	71,315,170 (41.1)	35,072,408 (43.8)	32,722,224 (47.7)	3,084,776 (47.4)	1,956,501 (45.8)
>80	54,991,737 (16.5)	29,734,629 (17.1)	12,750,662 (15.9)	10,572,012 (15.4)	1,050,250 (16.2)	884,184 (20.7)
Census region						
Northeast	52,724,321 (15.8)	28,388,204 (16.3)	10,055,425 (12.6)	12,602,174 (18.4)	1,437,669 (22.1)	240,849 (5.6)
Midwest	73,567,662 (22.1)	43,868,335 (25.3)	19,384,965 (24.2)	8,591,856 (12.5)	1,178,627 (18.1)	543,878 (12.7)
South	144,485,257 (43.4)	72,175,266 (41.6)	46,509,451 (58.1)	23,487,388 (34.2)	1,515,022 (23.3)	798,130 (18.7)
West	62,392,381 (18.7)	29,260,852 (16.8)	4,136,997 (5.2)	23,932,446 (34.9)	2,371,736 (36.5)	2,690,350 (63.0)
Reason for visit (by symptom cluster)						
General	66,792,161 (20.1)	30,514,486 (17.6)	16,784,380 (21.0)	17,076,980 (25.0)	1,663,937 (25.7)	752,378 (17.8)
Psychiatric	5,950,254 (1.8)	3,693,778 (2.1)	1,330,481 (1.7)	783,822 (1.1)	82,064 (1.3)	60,110 (1.4)
Neurological	12,546,663 (3.8)	6,881,087 (4.0)	2,981,361 (3.7)	2,349,128 (3.4)	193,330 (3.0)	141,757 (3.3)
Cardiovascular and Lymphatic	1,163,082 (0.4)	661,025 (0.4)	252,045 (0.3)	223,382 (0.3)	10,889 (0.2)	15,740 (0.4)
Eyes and Ears	19,043,040 (5.7)	9,467,954 (5.5)	4,780,233 (6.0)	4,250,476 (6.2)	300,945 (4.6)	243,432 (5.8)
Respiratory	57,172,175 (17.2)	26,829,823 (15.5)	16,686,052 (20.9)	11,664,756 (17.1)	1,118,061 (17.3)	873,482 (20.6)
Digestive	43,745,812 (13.2)	21,521,065 (12.4)	10,126,542 (12.7)	10,674,855 (15.6)	797,588 (12.3)	625,762 (14.8)
Genitourinary	7,913,120 (2.4)	3,721,422 (2.1)	2,247,735 (2.8)	1,779,917 (2.6)	95,201 (1.5)	68,844 (1.6)
Skin, Nails, and Hair	17,686,667 (5.3)	8,655,898 (5.0)	4,758,158 (6.0)	3,741,652 (5.5)	347,128 (5.4)	183,831 (4.3)
Musculoskeletal	27,378,747 (8.2)	16,509,157 (9.5)	5,906,982 (7.4)	4,121,511 (6.0)	495,275 (7.6)	345,822 (8.2)
Other	72,553,947 (21.9)	44,642,401 (25.8)	13,936,233 (17.5)	11,676,985 (17.1)	1,376,668 (21.2)	921,660 (21.8)

The missing proportion for arrival time, residency type, insurance, reason for visit and residency type is <5%; the missing proportion for temperature, heart rate, and blood pressure is 5–10%; the missing proportion for triage level is 20%, for Seen within last 72 h is 15%, for pain level is 49%. All p-values from chi-square tests are <0.001.

Tables 2, 3 and Supplement Table 3 summarize the main outcomes of interest across the sample and stratified by race. After adjusting for other covariates, Black patients and Hispanic patients were 8% less likely (aOR 0.92, CI 0.91–0.92), and 14% less likely (aOR 0.86, CI 0.86–0.86), respectively, than whites to receive immediate or emergent ESI score as opposed to semi- or non-urgent scores. Asian patients were more likely than whites to receive immediate or emergent (aOR 1.05, CI 1.04–1.0.5) or urgent care (aOR 1.15, CI 1.15–1.16) scores as opposed to semi- or non-urgent care needs in all models.

**Table 2:** Proportion of emergency severity index, hospital admission, ICU admission, medical resources utilization, stratified by race/ethnics, NHAMCS 2005–2016.

	**All**	**White**	**Black**	**Hispanic**	**Asian**	**Other**
ESI score						
Immediate	5,020,918 (1.8)	2,741,469 (1.9)	1,200,498 (1.8)	901,281 (1.6)	112,686 (2.0)	64,984 (1.9)
Emergent	21,346,130 (7.8)	12,062,886 (8.4)	4,870,376 (7.4)	3,723,519 (6.7)	441,215 (7.9)	248,133 (7.1)
Urgent	99,842,177 (36.3)	52,495,741 (36.4)	23,862,805 (36.3)	19,894,999 (35.6)	2,253,751 (40.4)	1,334,881 (38.2)
Semi-urgent	115,276,218 (41.9)	59,887,811 (41.5)	27,380,048 (41.7)	24,107,749 (43.1)	2,365,042 (42.4)	1,535,568 (43.9)
Non-urgent	33,470,812 (12.2)	17,076,986 (11.8)	8,406,217 (12.8)	7,263,184 (13.0)	411,097 (7.4)	313,328 (9.0)
Hospital admission	15,865,082 (4.8)	9,149,439 (5.3)	3,133,698 (3.9)	2,971,794 (4.3)	342,705 (5.3)	267,446 (6.3)
ICU	914,937 (0.3)	453,609 (0.3)	202,374 (0.3)	210,375 (0.3)	45,502 (0.7)	3,077 (0.1)
In hospital death	139,666 (0.0)	63,413 (0.0)	35,237 (0.0)	38,287 (0.1)	2,729 (0.0)	0 (0.0)
Blood test	58,141,100 (17.5)	32,166,587 (18.5)	12,315,970 (15.4)	11,792,576 (17.2)	1,097,368 (16.9)	768,599 (18.0)
Any image	108,114,905 (32.5)	62,043,795 (35.7)	23,385,877 (29.2)	19,350,391 (28.2)	2,049,570 (31.5)	1,285,272 (30.1)
X-ray	89,642,862 (26.9)	51,045,276 (29.4)	20,059,185 (25.0)	15,607,215 (22.7)	1,795,095 (27.6)	1,136,091 (26.6)
CT	19,123,492 (5.7)	12,132,915 (7.0)	3,365,450 (4.2)	3,205,937 (4.7)	236,894 (3.6)	182,296 (4.3)
Ultrasound	5,184,122 (1.6)	2,643,688 (1.5)	1,055,389 (1.3)	1,327,356 (1.9)	102,929 (1.6)	54,761 (1.3)
MRI	537,589 (0.2)	258,519 (0.1)	123,646 (0.2)	155,110 (0.2)	314 (0.0)	0 (0.0)
Other Image	1,909,039 (0.6)	1,009,607 (0.6)	477,377 (0.6)	362,436 (0.5)	33,549 (0.5)	26,070 (0.6)
Procedure	122,284,391 (36.7)	67,698,028 (39.0)	28,013,125 (35.0)	22,628,628 (33.0)	2,436,860 (37.5)	1,507,750 (35.3)
Waiting time [minutes, means (95% CI)]	46.1 (45.6–46.7)	42.0 (41.4–42.7)	50.5 (49.5–51.6)	52.0 (50.7–53.2)	44.0 (40.5–47.5)	40.9 (37.6–44.2)
Length of visit [minutes, means (95% CI)]	152.9 (151.6–154.1)	143.9 (142.2–145.6)	159.5 (157.0–161.9)	167.7 (164.4–170.9)	167.1 (159.2–175.0)	137.2 (129.5–144.9)

*Waiting time is time from arrival to seeing the physician; length of visit is time from arrival to discharge. All p-values from chi-square tests are <0.001*.

**Table 3:** Odds ratio of emergency severity index, hospital admission, ICU admission, medical resources utilization, stratified by race/ethnics, NHAMCS 2005–2016.

	**Race/ethnic group**	**Crude odds ratio**	**Adjusted for**			
			**Demographics**	**+ Social economic**	**+ Visiting & clinical**	**+ ESI score**
ESI Score: immediate or emergent vs. semi or non-urgent	White	Reference (1)	Reference (1)	Reference (1)	Reference (1)	
	Black	0.88 (0.88–0.88)	0.89 (0.89–0.89)	0.96 (0.96–0.96)	0.92 (0.91–0.92)	
	Hispanic	0.77 (0.77–0.77)	0.78 (0.78–0.78)	0.84 (0.84–0.85)	0.86 (0.86–0.86)	
	Asian	1.04 (1.03–1.04)	1.07 (1.07–1.07)	1.05 (1.05–1.05)	1.05 (1.04–1.05)	
	Other	0.88 (0.88–0.88)	0.88 (0.88–0.89)	0.95 (0.95–0.95)	0.94 (0.93–0.94)	
ESI score: urgent vs. semi or non-urgent	White	Reference (1)	Reference (1)	Reference (1)	Reference (1)	
	Black	0.98 (0.98–0.98)	0.99 (0.99–0.99)	1.03 (1.03–1.03)	1.00 (1.00–1.00)	
	Hispanic	0.93 (0.93–0.93)	0.95 (0.95–0.95)	0.98 (0.98–0.98)	0.94 (0.94–0.94)	
	Asian	1.19 (1.19–1.19)	1.23 (1.23–1.23)	1.21 (1.21–1.21)	1.15 (1.15–1.16)	
	Other	1.06 (1.06–1.06)	1.07 (1.07–1.07)	1.08 (1.08–1.08)	1.06 (1.06–1.07)	
Hospital admission	White	Reference (1)	Reference (1)	Reference (1)	Reference (1)	Reference (1)
	Black	0.73 (0.73–0.73)	0.74 (0.74–0.74)	0.74 (0.74–0.74)	0.69 (0.69–0.69)	0.72 (0.72–0.72)
	Hispanic	0.81 (0.81–0.82)	0.83 (0.83–0.83)	0.88 (0.88–0.88)	0.85 (0.84–0.85)	0.97 (0.97–0.97)
	Asian	1.00 (1.00–1.00)	1.03 (1.03–1.03)	1.05 (1.04–1.05)	0.95 (0.94–0.95)	1.08 (1.07–1.08)
	Other	1.20 (1.20–1.21)	1.18 (1.18–1.19)	1.34 (1.33–1.34)	1.21 (1.21–1.22)	1.43 (1.43–1.44)
ICU	White	Reference (1)	Reference (1)	Reference (1)	Reference (1)	Reference (1)
	Black	0.97 (0.96–0.97)	0.95 (0.95–0.96)	0.94 (0.93–0.94)	0.82 (0.81–0.82)	1.04 (1.03–1.05)
	Hispanic	1.18 (1.17–1.18)	1.15 (1.14–1.16)	1.22 (1.21–1.22)	1.17 (1.17–1.18)	1.21 (1.20–1.21)
	Asian	2.69 (2.67–2.72)	2.59 (2.57–2.62)	2.65 (2.62–2.67)	2.46 (2.43–2.48)	2.83 (2.80–2.86)
	Other	0.28 (0.27–0.29)	0.26 (0.25–0.27)	0.38 (0.37–0.39)	0.33 (0.31–0.34)	0.39 (0.37–0.40)
Blood test	White	Reference (1)	Reference (1)	Reference (1)	Reference (1)	Reference (1)
	Black	0.80 (0.80–0.80)	0.83 (0.83–0.83)	0.82 (0.82–0.82)	0.74 (0.74–0.74)	0.76 (0.76–0.76)
	Hispanic	0.91 (0.91–0.91)	1.00 (1.00–1.00)	1.05 (1.05–1.05)	0.90 (0.90–0.90)	0.96 (0.96–0.96)
	Asian	0.89 (0.89–0.90)	1.02 (1.02–1.02)	1.05 (1.05–1.05)	0.92 (0.92–0.92)	0.93 (0.92–0.93)
	Other	0.97 (0.96–0.97)	1.02 (1.02–1.03)	1.11 (1.11–1.12)	1.03 (1.03–1.04)	1.14 (1.14–1.14)
Any imaging	White	Reference (1)	Reference (1)	Reference (1)	Reference (1)	Reference (1)
	Black	0.74 (0.74–0.74)	0.78 (0.78–0.78)	0.80 (0.80–0.80)	0.83 (0.83–0.83)	0.83 (0.83–0.83)
	Hispanic	0.71 (0.71–0.71)	0.77 (0.77–0.77)	0.83 (0.83–0.83)	0.88 (0.88–0.88)	0.91 (0.90–0.91)
	Asian	0.83 (0.83–0.83)	0.94 (0.94–0.94)	0.96 (0.96–0.96)	0.93 (0.93–0.93)	0.92 (0.92–0.93)
	Other	0.77 (0.77–0.78)	0.82 (0.82–0.82)	0.87 (0.87–0.87)	0.83 (0.83–0.84)	0.84 (0.84–0.84)
X-ray	White	Reference (1)	Reference (1)	Reference (1)	Reference (1)	Reference (1)
	Black	0.80 (0.80–0.80)	0.83 (0.83–0.83)	0.84 (0.84–0.84)	0.88 (0.88–0.88)	0.86 (0.86–0.86)
	Hispanic	0.71 (0.71–0.71)	0.75 (0.75–0.75)	0.80 (0.80–0.80)	0.86 (0.85–0.86)	0.85 (0.85–0.85)
	Asian	0.92 (0.92–0.92)	1.00 (1.00–1.00)	1.03 (1.03–1.03)	0.99 (0.99–1.00)	0.98 (0.98–0.98)
	Other	0.87 (0.87–0.87)	0.91 (0.90–0.91)	0.96 (0.96–0.96)	0.94 (0.94–0.95)	0.94 (0.94–0.94)
CT	White	Reference (1)	Reference (1)	Reference (1)	Reference (1)	Reference (1)
	Black	0.58 (0.58–0.59)	0.63 (0.63–0.63)	0.68 (0.68–0.68)	0.69 (0.69–0.69)	0.72 (0.72–0.72)
	Hispanic	0.65 (0.65–0.65)	0.77 (0.77–0.77)	0.86 (0.86–0.86)	0.87 (0.87–0.87)	0.96 (0.96–0.96)
	Asian	0.50 (0.50–0.51)	0.62 (0.62–0.63)	0.64 (0.63–0.64)	0.62 (0.61–0.62)	0.58 (0.57–0.58)
	Other	0.59 (0.59–0.60)	0.66 (0.66–0.67)	0.72 (0.72–0.73)	0.65 (0.64–0.65)	0.76 (0.75–0.76)
Ultrasound	White	Reference (1)	Reference (1)	Reference (1)	Reference (1)	Reference (1)
	Black	0.86 (0.86–0.87)	0.92 (0.92–0.93)	0.98 (0.98–0.98)	0.91 (0.91–0.92)	1.04 (1.04–1.05)
	Hispanic	1.28 (1.27–1.28)	1.52 (1.52–1.53)	1.45 (1.45–1.46)	1.24 (1.24–1.24)	1.43 (1.43–1.44)
	Asian	1.04 (1.03–1.05)	1.37 (1.36–1.38)	1.28 (1.27–1.28)	1.31 (1.31–1.32)	1.23 (1.22–1.24)
	Other	0.84 (0.83–0.85)	0.96 (0.95–0.97)	0.90 (0.89–0.90)	0.86 (0.86–0.87)	1.05 (1.04–1.06)
MRI	White	Reference (1)	Reference (1)	Reference (1)	Reference (1)	Reference (1)
	Black	1.04 (1.03–1.04)	1.10 (1.09–1.10)	1.11 (1.10–1.12)	1.16 (1.16–1.17)	1.08 (1.07–1.09)
	Hispanic	1.52 (1.51–1.53)	1.71 (1.69–1.72)	1.89 (1.88–1.91)	2.04 (2.03–2.05)	2.35 (2.33–2.36)
	Asian	0.03 (0.03–0.04)	0.04 (0.03–0.04)	0.04 (0.04–0.04)	0.04 (0.03–0.04)	0.05 (0.04–0.05)
	Other	–	–	–	–	–
Procedure	White	Reference (1)	Reference (1)	Reference (1)	Reference (1)	Reference (1)
	Black	0.86 (0.86–0.86)	0.89 (0.89–0.89)	0.92 (0.92–0.92)	0.97 (0.97–0.98)	0.98 (0.98–0.98)
	Hispanic	0.82 (0.82–0.82)	0.87 (0.87–0.87)	0.90 (0.90–0.90)	0.95 (0.95–0.95)	0.97 (0.97–0.97)
	Asian	1.00 (1.00–1.00)	1.09 (1.09–1.09)	1.08 (1.08–1.08)	1.09 (1.09–1.10)	1.00 (1.00–1.01)
	Other	0.86 (0.86–0.86)	0.90 (0.90–0.90)	0.92 (0.92–0.93)	0.89 (0.89–0.89)	0.89 (0.89–0.89)

*+ Demographics adjusts for gender, age group; + Socioeconomic adjusts for residence type, insurance type, census region; + Visiting & Clinical: year, week of day, arrival by ambulance, seen within last 72 h, pain level, temperature, heart rate, dialytic blood pressure*.

After adjusting for other covariates (including ESI level), Blacks and Hispanics were also 28 and 3%, respectively, less likely than whites to be admitted to the hospital following their ED visit (aOR 0.72, CI 0.72–0.72; aOR 0.97, CI 0.97–0.97). Asian patients were 1.08 times more likely than whites to be admitted to the hospital following an ED visit (aOR 1.08, CI 1.07–1.08). Blacks and Hispanics were 1.04 and 1.21 times, respectively, more likely to receive ICU admission in the fully adjusted models (aOR 1.04, CI: 1.03–1.05; OR 1.21, CI 1.20–1.21).

After adjusting for other covariates (including ESI level), Black patients were 24% (aOR 0.76, CI 0.76–0.76) less likely to have a blood test during the ED visit than whites. Blacks, Hispanics and Asians were 17% (aOR 0.83, CI 0.83–0.83), 9% (aOR 0.91, CI 0.90–0.91), and 8% (aOR 0.92, CI 0.92–0.93) less likely to receive any imaging than whites. Specifically, Blacks, Hispanics and Asians were 28% (aOR 0.72 CI 0.72–0.72), 4% (aOR 0.96, CI 0.96–0.96), and 42% (aOR 0.92, CI 0.57–0.58) less likely to receive a CT scan as compared to white pediatric ED patients. However, Blacks, Hispanics, and Asians were 1.04 (aOR 1.04, CI 1.04–1.05), 1.43 (aOR 1.43, CI 1.43–1.44), and 1.23 (aOR 1.23, CI 1.22–1.24) times, respectively, more likely than whites to receive ultrasound. Relative to whites, Blacks and Hispanics were modestly less likely to receive general procedures (aOR 0.98, CI 0.98–0.98; and aOR 0.97, CI 0.97–0.97, respectively) while Asians were not (aOR 1.00, CI 1.00–1.01). After adjusting for other covariates, waiting times in the ED were significantly greater for Black and Hispanic children (*p* < 0.001) than for white children (Table 4).

**Table 4:** Linear regression between wait time or length of visit and by race/ethnics, NHAMCS 2005–2016.

	**Wait time**		**Length of visit**	
	**Beta (95% CI)**	***p*-value**	**Beta (95% CI)**	***p*-value**
White	Reference (1)		Reference (1)	
Black	0.190 (0.157–0.224)	<0.001	0.138 (0.123–0.152)	<0.001
Hispanic	0.178 (0.141–0.214)	<0.001	0.173 (0.157–0.189)	<0.001
Asian	−0.018 (−0.102–0.065)	0.665	0.010 (0.063–0.137)	<0.001
Other	0.068 (−0.046–0.183)	0.242	0.003 (−0.024–0.080)	0.287

*The model was adjusted for the following factors: demographics (gender, age group), socioeconomic (residence type, insurance type, census region), visiting and clinical (year, week of day, arrival by ambulance, seen within last 72 h, pain level, temperature, heart rate, dialytic blood pressure), and ESI score. The betas are the coefficients between the log (wait time or length of visit) and the racial/ethnic group*.

**Figure 1:**
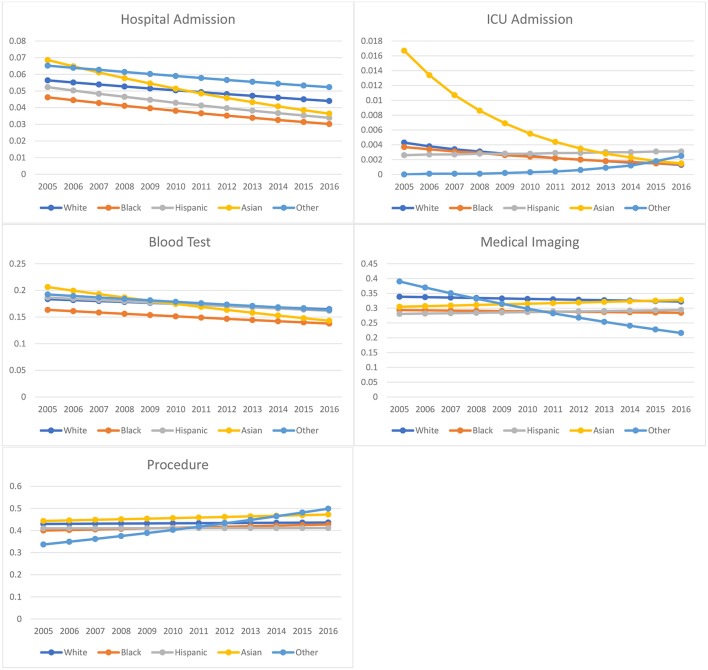
Racial/ethnic-specific ED health outcome and medical resource utilization rate from 2005 to 2016: NHAMCS 2005–2016.

Figure 1 displays trends in disposition and resource utilization outcomes over time (2005–2016) by racial group. Table 5 includes estimated rates and changes in rates over time for disposition and resource utilization outcomes. Rates of hospitalization significantly decreased over time in all racial groups. However, these rates decreased the least among whites as compared to other racial groups (*p* < 0.001). Of note, hospital admission for whites decreased by 21.99%, compared to 34.63 and 35.18% for Black and Hispanic patients, respectively. Rates of medical imaging utilization significantly decreased over time in whites and Blacks but increased in Hispanics and Asians. Rates of blood testing significantly decreased over time among all racial groups, but these rates decreased the least in white patients as compared to other racial groups (*p* < 0.001). Procedure utilization rates increased across all racial groups during the period of our study.

**Table 5:** Race/ethnicity-specific rates of disposition outcome and medical resources utilization: NHAMCS 2005–2016.

		Rate^*^		**Trend**	***p***	***p*^†^**
**Outcome**	**Race/ethnicity**	**2005**	**2016**	**2005–2016**	**trend**	**Race/ethnic difference in trend**
Hospital Admission	White	0.0564	0.044	−21.99%	<0.001	
	Black	0.0462	0.0302	−34.63%	<0.001	<0.001
	Hispanic	0.0523	0.0339	−35.18%	<0.001	<0.001
	Asian	0.0686	0.0364	−46.94%	<0.001	<0.001
	Other	0.0652	0.0523	−19.79%	<0.001	<0.001
ICU Admission	White	0.0043	0.0013	−69.77%	<0.001	
	Black	0.0037	0.0014	−62.16%	<0.001	<0.001
	Hispanic	0.0026	0.0031	19.23%	<0.001	<0.001
	Asian	0.0167	0.0015	−91.02%	<0.001	<0.001
	Other	–	0.0025		<0.001	<0.001
Blood test	White	0.1836	0.1647	−10.29%	<0.001	
	Black	0.1635	0.1378	−15.72%	<0.001	<0.001
	Hispanic	0.1867	0.1618	−13.34%	<0.001	<0.001
	Asian	0.2063	0.1431	−30.63%	<0.001	<0.001
	Other	0.1924	0.1633	−15.12%	<0.001	<0.001
Any Imaging	White	0.3387	0.3224	−4.81%	<0.001	
	Black	0.2935	0.2844	−3.10%	<0.001	<0.001
	Hispanic	0.2806	0.2941	4.81%	<0.001	<0.001
	Asian	0.3048	0.3275	7.45%	<0.001	<0.001
	Other	0.3902	0.2161	−44.62%	<0.001	<0.001
Procedure	White	0.4298	0.4362	1.49%	<0.001	
	Black	0.3998	0.4272	6.85%	<0.001	<0.001
	Hispanic	0.4099	0.4114	0.37%	<0.001	<0.001
	Asian	0.4432	0.4723	6.57%	<0.001	<0.001
	Other	0.3369	0.4988	48.06%	<0.001	<0.001

* Predicted rate and trend were derived from a model using data over the time period, modeling time as a linear trend.

† *From the time by Race/ethnicity interaction in the Poisson regression model*.

## Discussion

We examined differences in many aspects of pediatric ED evaluation, management, disposition, throughput, and resource utilization among racial groups and explored trends in these measures over time. Our analysis responds to documented shortcomings in the pediatric racial health disparities literature by including a meaningful comparison group (whites), analyzing three racial minority groups rather than a combined non-white group, adjusting for likely confounding social and demographic factors, and focusing on multiple aspects of the high-stakes arena of emergency care (1). Across our analyses, we observed significant racial differences in ED encounters and treatment for pediatric patients between 2005 and 2016. We discuss these findings within two broad categories: characteristics of the ED visit and care received in the ED.

### Characteristics of ED Visit

Black and Hispanic children were less likely than whites to be classified as needing immediate or emergent care as opposed to semi- or non-urgent care, which is consistent with prior literature (6–9). This difference could not be fully explained by possible confounding factors available in the dataset, such as demographic, socioeconomic, or clinical variables. An epidemiological study of an urban ED (Baltimore, Maryland) found that Black children were more likely than whites to be brought to the ED for non-urgent care needs, the authors positing that Black families' greater proximity to the ED may be a primary cause of the disparity (10). Similarly, a study of predominately Black (96%) caregivers in an urban setting (New Orleans, LA) found that one-third routinely brought their children to the ED for non-urgent acute illness (11). The authors reported that the ED's shorter wait and discharge times were the foremost motivation for this care-seeking pattern (11). More research is needed that captures patient-level metrics of the distance, time of travel, mode of transportation, and actual or perceived advantages of ED vs. non-ED care options in the local context, in order to properly adjust for such confounders.

Our analysis also revealed that Asian children were more likely than whites to present to the ED for needs classified as immediate or emergent, which marks a divergence from the other racial minority groups in our sample that cannot be adequately explained by other socioeconomic or clinical characteristics. However, Asian children have been found to have better health profiles as compared to other racial groups in the US, which could have a significant influence on their use of and treatment in the healthcare system (3). However, the literature on health disparities among Asian children in the US remains sparse, and thus more research is needed in various healthcare settings to identify possible contributing factors.

Relative to Asians and whites, Blacks and Hispanics showed a lower likelihood of being admitted to the hospital following an ED visit, even after adjustment for clinical factors and vital signs in addition to social and demographic measures. Our plot of predicted rates of hospitalization over the 12-years span (2005–2016) reveals that racial minority groups are experiencing more significant declines in hospitalization rates as compared to whites, although hospitalization is trending downward for all groups.

The pattern of admissions to the ICU diverged slightly, with only Black children less likely than whites to be admitted to the ICU, while Hispanic and Asian children showed higher odds of ICU admission than whites. In terms of 12-years trends, we see declines in the predicted rates of ICU admission for Asian, white, and Black children, while rates have increased over time for Hispanic children (note that high ICU admission rates for Asian children near the start of the 2005–2016 period are likely spurious results, affected by small sample sizes). In the fully adjusted linear regression models, Hispanic and Black children were predicted to have significantly longer wait times in the ED as compared to whites, as well as longer visit times overall. The finding for Hispanic children is consistent with a previous study on NHAMCS-ED data from 1997 to 2000, while the longer wait and visit times for Black children were not significant in the earlier dataset with a smaller sample size (12). Compared to whites, Asian children did not have significantly different wait times but did have slightly greater overall length of ED stay.

### Care Received in the ED

Our analysis also focused on the administration of tests, imaging, and general procedures during children's visits to the ED. The patterns in the medical resource utilization aspect of ED care varied to a greater degree across the racial minority groups. For example, Black, Hispanic, and Asian children were significantly less likely than whites to receive blood tests, X-rays, and CT scans, but Blacks and Hispanics were more likely than whites to receive MRI scans. While finer-grained information is not available, the receipt of general procedures was slightly lower in Black and Hispanic relative to white children. In contrast, Asians were as likely as whites to receive general procedures.

In terms of 12-years trends, the rates of medical imaging utilization decreased slightly but significantly for whites and Blacks, while rates increased slightly for Hispanics and Asians. Rates of blood tests significantly decreased over time for all racial groups, but these rates decreased the least among white children. Utilization rates for general procedures also increased across all racial groups.

## Limitations

A major limitation of our study is the potential for sampling biases and errors in the NHAMCS-ED data. Namely, heterogeneity in documentation (e.g., due to differences in electronic health records practices) may involve abstraction errors, missing responses, and inaccurate responses. However, such systematic biases should not moderate, in any consistent way, the statistical associations reported herein. Additionally, 6% of the total NHAMCS-ED sample was removed for not having a documented race/ethnicity; however, this figure is below the acceptable non-response threshold for this type of data source (13) and thus does not invalidate the primary exposure variable for this analysis. Another limitation of our study is our use of system-based reasons for ED visit (e.g., “respiratory”) as units of analysis in our model rather specific complaints (e.g., “shortness of breath”). Future investigations of disparities in ED outcomes and resource utilization for more specific reasons for visit could be revealing.

## Conclusions

Our study revealed disparities across multiple dimensions of the ED visits and care received by Black and Hispanic pediatric patients in the US, while Asian children did not experience similar disparities. Our analysis cannot determine the extent to which the observed disparities owe to bias on the part of ED staff and care providers. Understanding the role of personnel bias remains a topic for future research and is particularly pressing for the development of institutional correctives. To inform public policy, further research is needed to identify other underlying causes as well as the long-term health consequences of the observed racial disparities in pediatric emergency care, especially in light of the shift toward greater representation of racial minorities in this age group.

## Data Availability Statement

The NHAMCS-ED dataset can be accessed through the website of the US Centers for Disease Control and Prevention (CDC) (https://www.cdc.gov/nchs/ahcd/index.htm).

## Ethics Statement

This study used pre-existing, de-identified data and was categorized as exempt from ethical approval by the University of Michigan's Institutional Review Board.

## Author Contributions

XZ had full access to all the data in the study and takes responsibility for the integrity of the data and the accuracy of the data analysis. XZ and PM: concept and design. MC, XZ, and TH: drafting of the manuscript. KH, CF, and PM: critical revision of the manuscript for important intellectual content. XZ: statistical analysis. XZ and PM: obtained funding. XZ: administrative, technical, or material support. XZ and PM: supervision. All authors: acquisition, analysis, or interpretation of data.

### Conflict of Interest

The authors declare that the research was conducted in the absence of any commercial or financial relationships that could be construed as a potential conflict of interest.
