# Supplementary material for: Racial and Ethnic Disparities in Emergency Department Care and Health Outcomes Among Children in the United States
Source: Front Pediatr. 2019 Dec 19;7:525. doi: 10.3389/fped.2019.00525 (PMC6951392; doi:10.3389/fped.2019.00525)
Supplement: Supplementary file 1 [file Table_1.docx]

Supplement Table 1. Types of Procedures Collected in the NHAMCS 2005–2016

| Year | Procedure |
| --- | --- |
| 2012–2016 | BiPAP/CPAP; Bladder catheter; Cast, splint, wrap; Central line Other; IV fluids; CPR; Endotracheal intubation; Incision & drainage (I&D); IV fluids; Lumbar puncture (LP); Nebulizer therapy; Pelvic exam; Skin adhesives; Suturing/Staples; Other |
| 2007–2011 | IV fluids; Cast; splint or wrap; Suturing/Staples; Incision & drainage (I&D); Foreign body removal; Nebulizer therapy; Bladder catheter; Pelvic exam; Central line; CPR; Endotracheal intubation; Other |
| 2005–2006 | Bladder catheter; CPR; Endotracheal intubation; IV fluids; Nebulizer therapy; NG tube/gastric suction; OB/GYN care; orthopedic care; Thrombolytic therapy; Wound care; Other |

Supplement Table 2. Baseline characteristics of patients presenting to the ED, stratified by race/ethnics, NHAMCS 2005–2016 (Unweighted Sample)

|  | All | White | Black | Hispanic | Asian | Other |
| --- | --- | --- | --- | --- | --- | --- |
|  | 78,471 | 39,274 (50.1) | 19,571 (24.9) | 16,534 (21.1) | 2,035 (2.6) | 1,057 (1.4) |
| Male | 40,828(52.0) | 20,461(52.1) | 9,994(51.1) | 8,676(52.5) | 1,132(55.6) | 565(53.5) |
| Age |  |  |  |  |  |  |
| 0-<1 | 9,394(12.0) | 3,950(10.1) | 2,500(12.8) | 2,485(15.0) | 288(14.2) | 171(16.2) |
| 1-<6 | 26,958(34.4) | 12,422(31.6) | 6,871(35.1) | 6,396(38.7) | 869(42.7) | 400(37.8) |
| 6-<12 | 16,966(21.6) | 8,655(22.0) | 4,136(21.1) | 3,567(21.6) | 410(20.1) | 198(18.7) |
| 12-18 | 25,153(32.1) | 14,247(36.3) | 6,064(31.0) | 4,086(24.7) | 468(23.0) | 288(27.2) |
| Residence type |  |  |  |  |  |  |
| Private residence | 75,397(99.0) | 37,756(98.9) | 18,771(99.0) | 15,883(99.3) | 1,959(99.0) | 1,028(99.1) |
| Nursing home | 84(0.1) | 38(0.1) | 22(0.1) | 19(0.1) | 5(0.3) | 0(0.0) |
| Homeless | 63(0.1) | 31(0.1) | 19(0.1) | 8(0.1) | 2(0.1) | 3(0.3) |
| Other | 603(0.8) | 338(0.9) | 156(0.8) | 90(0.6) | 13(0.7) | 6(0.6) |
| Insurance type |  |  |  |  |  |  |
| Private insurance | 25,699(34.5) | 17,239(46.0) | 4,055(21.9) | 3,228(20.6) | 845(43.9) | 332(32.9) |
| Medicare | 664(0.9) | 319(0.9) | 194(1.0) | 135(0.9) | 13(0.7) | 3(0.3) |
| Medicaid or CHIP | 40,357(54.1) | 16,271(43.4) | 12,216(66.1) | 10,396(66.3) | 892(46.4) | 582(57.6) |
| Uninsured | 6,201(8.3) | 2,780(7.4) | 1,655(9.0) | 1,579(10.1) | 124(6.4) | 63(6.2) |
| Other | 1,663(2.2) | 864(2.3) | 371(2.0) | 349(2.2) | 49(2.5) | 30(3.0) |
| Year |  |  |  |  |  |  |
| 2005 | 8,582(10.9) | 4,391(11.2) | 2,057(10.5) | 1,818(11.0) | 216(10.6) | 100(9.5) |
| 2006 | 9,712(12.4) | 4,828(12.3) | 2,490(12.7) | 1,960(11.9) | 298(14.6) | 136(12.9) |
| 2007 | 7,535(9.6) | 3,897(9.9) | 1,855(9.5) | 1,512(9.1) | 185(9.1) | 86(8.1) |
| 2008 | 6,836(8.7) | 3,480(8.9) | 1,913(9.8) | 1,195(7.2) | 166(8.2) | 82(7.8) |
| 2009 | 7,897(10.1) | 3,861(9.8) | 1,990(10.2) | 1,708(10.3) | 215(10.6) | 123(11.6) |
| 2010 | 7,808(10.0) | 3,836(9.8) | 1,863(9.5) | 1,785(10.8) | 203(10.0) | 121(11.4) |
| 2011 | 6,420(8.2) | 3,109(7.9) | 1,746(8.9) | 1,254(7.6) | 195(9.6) | 116(11.0) |
| 2012 | 5,759(7.3) | 2,916(7.4) | 1,314(6.7) | 1,360(8.2) | 115(5.7) | 54(5.1) |
| 2013 | 5,027(6.4) | 2,411(6.1) | 1,278(6.5) | 1,142(6.9) | 135(6.6) | 61(5.8) |
| 2014 | 5,101(6.5) | 2,523(6.4) | 1,227(6.3) | 1,164(7.0) | 112(5.5) | 75(7.1) |
| 2015 | 4,044(5.2) | 2,103(5.4) | 934(4.8) | 876(5.3) | 85(4.2) | 46(4.4) |
| 2016 | 3,750(4.8) | 1,919(4.9) | 904(4.6) | 760(4.6) | 110(5.4) | 57(5.4) |
| Day of Week |  |  |  |  |  |  |
| Sunday | 12,159(15.5) | 6,302(16.0) | 2,853(14.6) | 2,562(15.5) | 293(14.4) | 149(14.1) |
| Monday | 11,968(15.3) | 5,856(14.9) | 3,074(15.7) | 2,554(15.4) | 323(15.9) | 161(15.2) |
| Tuesday | 11,079(14.1) | 5,417(13.8) | 2,895(14.8) | 2,329(14.1) | 277(13.6) | 161(15.2) |
| Wednesday | 10,736(13.7) | 5,215(13.3) | 2,836(14.5) | 2,257(13.7) | 278(13.7) | 150(14.2) |
| Thursday | 10,726(13.7) | 5,363(13.7) | 2,680(13.7) | 2,254(13.6) | 279(13.7) | 150(14.2) |
| Friday | 10,416(13.3) | 5,139(13.1) | 2,573(13.1) | 2,294(13.9) | 269(13.2) | 141(13.3) |
| Saturday | 11,387(14.5) | 5,982(15.2) | 2,660(13.6) | 2,284(13.8) | 316(15.5) | 145(13.7) |
| Arrive by ambulance | 5,120(6.7) | 2,380(6.2) | 1,525(8.0) | 991(6.2) | 149(7.4) | 75(7.2) |
| Seen within last 72 hours | 2,413(3.5) | 1,090(3.2) | 554(3.3) | 663(4.7) | 61(3.3) | 45(4.7) |
| Pain level |  |  |  |  |  |  |
| No pain | 20,624(39.2) | 9,070(34.2) | 5,997(44.4) | 4,561(43.5) | 648(46.6) | 348(46.1) |
| Mild | 8,731(16.6) | 4,527(17.1) | 2,132(15.8) | 1,725(16.5) | 254(18.3) | 93(12.3) |
| Moderate | 14,628(27.8) | 8,165(30.8) | 3,272(24.2) | 2,642(25.2) | 353(25.4) | 196(26.0) |
| Severe | 8,673(16.5) | 4,750(17.9) | 2,111(15.6) | 1,558(14.9) | 136(9.8) | 118(15.6) |
| Temperature |  |  |  |  |  |  |
| 36 C-38 C | 61,177(83.0) | 31,282(84.7) | 15,133(82.5) | 12,430(80.1) | 1,521(79.6) | 811(80.6) |
| <=36 C | 3,247(4.4) | 1,790(4.8) | 791(4.3) | 575(3.7) | 59(3.1) | 32(3.2) |
| >38 C | 9,314(12.6) | 3,879(10.5) | 2,422(13.2) | 2,519(16.2) | 331(17.3) | 163(16.2) |
| Heart Rate |  |  |  |  |  |  |
| <=90 | 27,669(35.3) | 14,684(37.4) | 7,034(35.9) | 5,014(30.3) | 623(30.6) | 314(29.7) |
| 90-100 | 10,186(13.0) | 5,451(13.9) | 2,440(12.5) | 1,958(11.8) | 225(11.1) | 112(10.6) |
| 100-110 | 8,551(10.9) | 4,477(11.4) | 2,178(11.1) | 1,603(9.7) | 204(10.0) | 89(8.4) |
| 110-120 | 8,041(10.2) | 4,002(10.2) | 1,989(10.2) | 1,742(10.5) | 184(9.0) | 124(11.7) |
| >120 | 24,024(30.6) | 10,660(27.1) | 5,930(30.3) | 6,217(37.6) | 799(39.3) | 418(39.5) |
| DBP |  |  |  |  |  |  |
| <60 | 31,435(40.1) | 16,440(41.9) | 7,921(40.5) | 5,958(36.0) | 755(37.1) | 361(34.2) |
| 60-80 | 32,481(41.4) | 15,360(39.1) | 8,205(41.9) | 7,583(45.9) | 866(42.6) | 467(44.2) |
| >80 | 14,555(18.5) | 7,474(19.0) | 3,445(17.6) | 2,993(18.1) | 414(20.3) | 229(21.7) |
| Census Region |  |  |  |  |  |  |
| Northeast | 16,465(21.0) | 8,182(20.8) | 3,662(18.7) | 3,956(23.9) | 564(27.7) | 101(9.6) |
| Midwest | 17,764(22.6) | 10,919(27.8) | 4,331(22.1) | 2,202(13.3) | 236(11.6) | 76(7.2) |
| South | 29,996(38.2) | 14,246(36.3) | 10,521(53.8) | 4,706(28.5) | 321(15.8) | 202(19.1) |
| West | 14,246(18.2) | 5,927(15.1) | 1,057(5.4) | 5,670(34.3) | 914(44.9) | 678(64.1) |
| Reason for visit |  |  |  |  |  |  |
| General Symptoms | 15,778(20.2) | 6,896(17.6) | 4,044(20.8) | 4,116(25.0) | 512(25.2) | 210(20.0) |
| Symptoms Referable to Psychological and Mental Disorders | 1,534(2.0) | 838(2.1) | 364(1.9) | 285(1.7) | 34(1.7) | 13(1.2) |
| Symptoms Referable to the Nervous System | 2,835(3.6) | 1,491(3.8) | 704(3.6) | 545(3.3) | 65(3.2) | 30(2.9) |
| Symptoms Referable to the Cardiovascular and Lymphatic Systems | 241(0.3) | 143(0.4) | 55(0.3) | 37(0.2) | 4(0.2) | 2(0.2) |
| Symptoms Referable to the Eyes and Ears | 4,558(5.8) | 2,175(5.6) | 1,203(6.2) | 1,030(6.3) | 95(4.7) | 55(5.2) |
| Symptoms Referable to the Respiratory System | 13,127(16.8) | 5,853(15.0) | 3,905(20.0) | 2,836(17.2) | 324(16.0) | 209(19.9) |
| Symptoms Referable to the Digestive System | 10,189(13.0) | 4,834(12.4) | 2,436(12.5) | 2,494(15.2) | 259(12.8) | 166(15.8) |
| Symptoms Referable to the Genitourinary System | 1,959(2.5) | 873(2.2) | 571(2.9) | 455(2.8) | 40(2.0) | 20(1.9) |
| Symptoms Referable to the Skin, Nails, and Hair | 4,291(5.5) | 1,937(5.0) | 1,287(6.6) | 899(5.5) | 116(5.7) | 52(5.0) |
| Symptoms Referable to the Musculoskeletal System | 6,592(8.4) | 3,894(10.0) | 1,438(7.4) | 1,033(6.3) | 147(7.2) | 80(7.6) |
| Other | 17,042(21.8) | 10,186(26.0) | 3,482(17.9) | 2,729(16.6) | 433(21.3) | 212(20.2) |

Supplement Table 3. Proportion of Emergency Severity Index, Hospital admission, ICU admission, Medical resources utilization, stratified by race/ethnics, NHAMCS 2005–2016 (Unweighted Sample)

|  | All | White | Black | Hispanic | Asian | Other |
| --- | --- | --- | --- | --- | --- | --- |
| ESI score |  |  |  |  |  |  |
| Immediate | 1,360(2.1) | 753(2.3) | 340(2.1) | 220(1.6) | 33(1.9) | 14(1.6) |
| Emergent | 5,561(8.5) | 2,914(8.8) | 1,348(8.2) | 1,068(8.0) | 169(9.7) | 62(7.2) |
| Urgent | 24,585(37.6) | 12,324(37.4) | 6,081(37.0) | 5,090(38.1) | 742(42.6) | 348(40.2) |
| Semi-urgent | 25,746(39.4) | 12,932(39.2) | 6,498(39.6) | 5,304(39.7) | 643(36.9) | 369(42.6) |
| Non-urgent | 8,133(12.4) | 4,064(12.3) | 2,149(13.1) | 1,693(12.7) | 154(8.8) | 73(8.4) |
| Hospital Admission | 4,087(5.2) | 2,215(5.6) | 868(4.4) | 830(5.0) | 113(5.6) | 61(5.8) |
| ICU | 248(0.3) | 115(0.3) | 59(0.3) | 55(0.3) | 17(0.8) | 2(0.2) |
| In hospital death | 33(0.0) | 16(0.0) | 10(0.1) | 6(0.0) | 1(0.0) | 0(0.0) |
| Blood test | 14,062(17.9) | 7,294(18.6) | 3,183(16.3) | 3,011(18.2) | 399(19.6) | 175(16.6) |
| Any image | 24,833(31.6) | 13,920(35.4) | 5,420(27.7) | 4,555(27.5) | 607(29.8) | 331(31.3) |
| X-ray | 20,731(26.4) | 11,526(29.3) | 4,670(23.9) | 3,732(22.6) | 514(25.3) | 289(27.3) |
| CT | 4,216(5.4) | 2,616(6.7) | 748(3.8) | 720(4.4) | 84(4.1) | 48(4.5) |
| Ultrasound | 1,203(1.5) | 571(1.5) | 249(1.3) | 331(2.0) | 39(1.9) | 13(1.2) |
| MRI | 154(0.2) | 74(0.2) | 42(0.2) | 36(0.2) | 2(0.1) | 0(0.0) |
| Other Image | 500(0.6) | 229(0.6) | 125(0.6) | 128(0.8) | 14(0.7) | 4(0.4) |
| Procedure | 28,761(36.7) | 15,369(39.1) | 6,738(34.4) | 5,476(33.1) | 788(38.7) | 390(36.9) |
| Waiting time (minutes, means (95% CI)) | 48.2(47.7-48.7) | 43.0(42.3-43.7) | 53.5(52.4-54.6) | 54.8(53.4-56.1) | 44.8(41.2-48.4) | 45.5(42.0-49.1) |
| Length of visit (minutes, means (95% CI)) | 159.7(158.2-161.1) | 145.1(143.3-146.8) | 168.6(165.7-171.6) | 184.3(180.3-188.3) | 167.4(157.8-177.1) | 144.8(133.9-155.7) |
